# Neuroinflammation in dementia: A meta-analysis of PET imaging studies

**DOI:** 10.1097/MD.0000000000038086

**Published:** 2024-05-03

**Authors:** Jie Pan, Jin Hu, Danyang Meng, Liang Chen, Xianling Wei

**Affiliations:** a Department of Neurology, The First Hospital of Jiaxing (Affiliated Hospital of Jiaxing University), Jiaxing City, Zhejiang Province, China; b Department of Head and Neck Surgery, The First Hospital of Jiaxing (Affiliated Hospital of Jiaxing University), Jiaxing City, Zhejiang Province, China; c Department of Nuclear Medicine, The First Hospital of Jiaxing (Affiliated Hospital of Jiaxing University), Jiaxing City, Zhejiang Province, China.

**Keywords:** dementia, meta-analysis, neuroinflammation, positron emission tomography (PET), translocator protein (TSPO)

## Abstract

**Background:**

Dementia is a major public health challenge for aging societies worldwide. Neuroinflammation is thought to be a key factor in dementia development. The aim of this study was to comprehensively assess translocator protein (TSPO) expression by positron emission tomography (PET) imaging to reveal the characteristics of neuroinflammation in dementia.

**Methods:**

We used a meta-analysis to retrieve literature on TSPO expression in dementia using PET imaging technology, including but not limited to the quality of the study design, sample size, and the type of TSPO ligand used in the study. For the included studies, we extracted key data, including TSPO expression levels, clinical characteristics of the study participants, and specific information on brain regions. Meta-analysis was performed using R software to assess the relationship between TSPO expression and dementia.

**Results:**

After screening, 12 studies that met the criteria were included. The results of the meta-analysis showed that the expression level of TSPO was significantly elevated in patients with dementia, especially in the hippocampal region. The OR in the hippocampus was 1.50 with a 95% CI of 1.09 to 1.25, indicating a significant increase in the expression of TSPO in this region compared to controls. Elevated levels of inflammation in the prefrontal lobe and cingulate gyrus are associated with cognitive impairment in patients. This was despite an OR of 1.00 in the anterior cingulate gyrus, indicating that TSPO expression in this region did not correlate significantly with the findings. The overall heterogeneity test showed *I*² = 51%, indicating moderate heterogeneity.

**Conclusion:**

This study summarizes the existing literature on TSPO expression in specific regions of the brain in patients with dementia, and also provides some preliminary evidence on the possible association between neuroinflammation and dementia. However, the heterogeneity of results and limitations of the study suggest that we need to interpret these findings with caution. Future studies need to adopt a more rigorous and consistent methodological design to more accurately assess the role of neuroinflammation in dementia, thereby providing a more reliable evidence base for understanding pathological mechanisms and developing potential therapeutic strategies.

## 1. Introduction

Dementia has become a major public health challenge for today society, especially in the context of the increasing trend of global population aging.^[1]^ As an umbrella term for a range of neurodegenerative diseases, dementia includes various forms, such as Alzheimer disease, vascular dementia, and dementia with Lewy bodies.^[2–4]^ The common feature is the persistent decline in cognitive function and the gradual loss of the ability to perform daily living tasks. The quality of life of patients and their families is seriously affected. As research has progressed, neuroinflammation has been widely recognized as 1 of the key pathological mechanisms in the development of dementia. Not only does it drive the development of neurodegenerative changes in the early stages of the disease, but the neuroinflammatory response may be further exacerbated as the disease progresses, creating a vicious cycle.^[5]^

The development of positron emission tomography (PET) imaging has provided a powerful tool in the diagnosis and study of dementia.^[6]^ Through the use of radiolabeled TSPO, PET imaging is able to quantify, in vivo, the activation of microglia in the brain, a process that plays a central role in the neuroinflammatory response.^[7,8]^ Microglial cell activation is thought to be strongly correlated with the neuropathological features of dementia, and the level of TSPO expression positively correlates with the degree of inflammation.^[9,10]^ Thus, PET imaging provides us with a powerful means of assessing neuroinflammation and exploring its relationship with cognitive decline. However, TSPO imaging is fraught with uncertainty. Despite the interest in the role of TSPO in neuroinflammation, its widespread expression in healthy tissues, uncertain biological role, off-target binding in white matter, and low to moderate brain uptake of all reported radioligands limit its validity as a marker of neuroinflammation. These issues suggest that future neuroinflammatory imaging efforts will require a more precise and specific target than TSPO.

Although single PET imaging studies have made technological breakthroughs, their results have certain variability and limitations due to a variety of factors such as study design, sample size, and analysis methods.^[11–14]^ Furthermore, the overall understanding of PET studies in dementia remains limited, including the association of PET imaging with key indicators such as dementia-related biomarkers and survival time. An understanding of these correlations is essential for assessing the utility of PET imaging in clinical practice. To overcome the limitations of these individual studies, we systematically integrated the results of PET imaging studies on neuroinflammation in dementia from the existing literature by means of meta-analysis. The present study comprehensively considered the heterogeneity of the studies and aimed to reveal the overall distribution pattern of neuroinflammation in dementia and its correlation with the clinical manifestations of the disease, thus providing a more precise and comprehensive understanding of the pathophysiology. Through in-depth analysis of the available research data, we expect to not only improve our understanding of the mechanism of action of neuroinflammation in dementia, but also to identify potential biomarkers and provide new strategies for clinical diagnosis and treatment. In addition, our study also aims to explore the interactions between neuroinflammation and other pathological changes in dementia, as well as their potential roles in disease prevention and intervention, thus providing a scientific basis for future research directions in this field.

## 2. Materials and methods

### 2.1. Literature search

The literature search strategy employed in this study aimed to ensure coverage of all relevant research literature in order to provide a comprehensive understanding and assessment of neuroinflammation in dementia. To this end, an exhaustive search was conducted in major biomedical and scientific databases such as PubMed, Embase, Web of Science, The Cochrane Library and EBSCO. The search terms consisted of disease conditions (“Dementia” OR “Alzheimer Disease” OR “Vascular Dementia” OR “Lewy Body Dementia” OR “Frontotemporal Dementia”), pathological processes (“Neuroinflammation” OR “Brain Inflammation” OR “Microglial Activation” OR “Astrocytosis”), and imaging techniques (“Positron Emission Tomography” OR “PET Imaging” OR “Fluorodeoxyglucose FDG” OR “Pittsburgh Compound B” OR “Amyloid PET” OR “Tau PET”) were composed of related terms. In addition, we performed a manual search including relevant literature citations and abstracts of important academic conferences to minimize the risk of missing important studies.

### 2.2. Inclusion and exclusion criteria

Inclusion criteria were as follows: studies using PET technology; included studies had to involve patients with dementia, which included types such as Alzheimer disease, vascular dementia, dementia with Lewy bodies, and frontotemporal lobe dementia; and studies that included neuroinflammation-related markers (e.g., TSPO) for assessment.

Exclusion criteria included all non-English language literature; case reports, review articles, conference abstracts, and non-peer-reviewed publications; studies with major flaws in methodological design or incomplete data reporting.

### 2.3. Data extraction

Data extraction centered on several core elements: study design, sample size, baseline subject characteristics, type of PET tracer used, and the primary outcome of the study. We recorded baseline information such as the total sample size, age and gender of the participants, and the PET tracer used for each study, and to ensure the accuracy and consistency of the data, this process was carried out independently by 2 researchers with a standardized data extraction form. Any disagreements that arose during the extraction process were resolved through team discussions.

### 2.4. Evaluation of literature quality

In this meta-analysis of PET imaging studies of neuroinflammation in dementia, in order to ensure the quality of the included literature, we used the Newcastle-Ottawa Scale (NOS) for the evaluation of the quality of the literature. The NOS scale is a widely recognized tool for evaluating the quality of observational studies, including case–control studies and cohort studies. The scale is based on 3 main dimensions: selection of studies (maximum 4 points), fairness of comparisons (maximum 2 points), and reliability and validity of results (maximum 3 points). The total score is 9, with higher scores representing higher quality studies. Each document was scored independently by 2 researchers to minimize subjective bias. Disagreements that arose during the scoring process were resolved after team discussion or consultation with third-party experts.

### 2.5. Statistical analysis

In this meta-analysis, we used a variety of statistical methods to comprehensively analyze the collected data. Inhibitory analysis was performed using the *I*² statistic test, which quantifies the proportion of heterogeneity, with higher values indicating greater inter-study variability, and *I*^2^ > 50% indicates greater heterogeneity in the included literature. A random effects model was used, and the combined effect sizes used Odds Risk and the corresponding 95% confidence interval (CI). Sensitivity analyses were performed by recalculating the combined effect sizes after excluding a study one-by-one, aiming to test the robustness of our results. Publication bias was assessed using funnel plot and Egger and Bgger tests. The funnel plot allows for a visual determination of the presence of bias, while Egger test provides a statistical test. Finally, all statistical analyses were performed using R4.3.1 and all statistical results were considered statistically significant at *P* < .05.

## 3. Results

### 3.1. Literature screening results and basic characteristics

**Figure 1.**
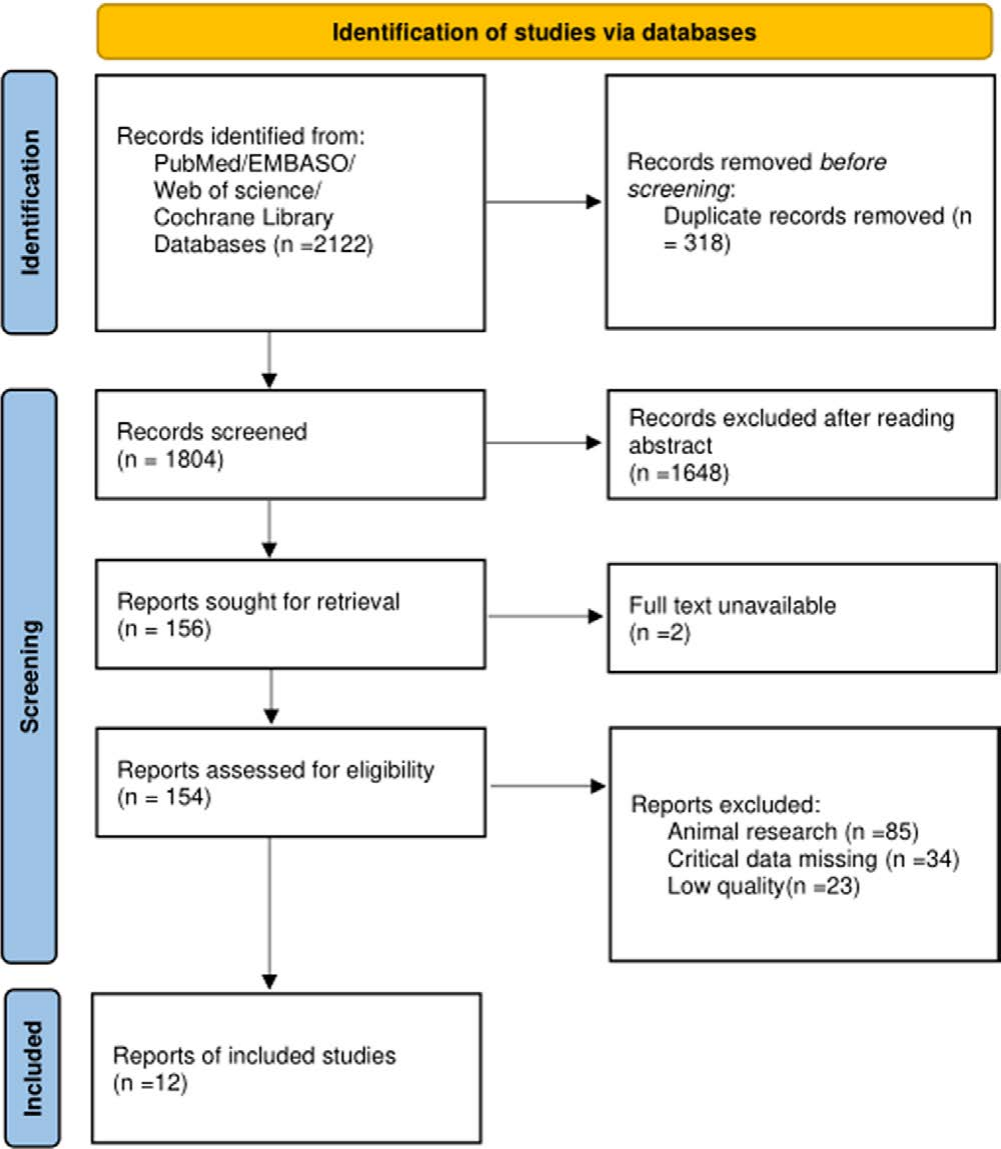
Schematic diagram of the screening process for inclusion of literature and results.

After a preliminary database search, we obtained a total of 2122 potentially relevant literature. After eliminating the literature that did not meet the inclusion criteria through the initial screening, we conducted a meticulous full-text review of the remaining 157 papers to ensure that the selected studies strictly met our inclusion guidelines. The final inclusion of 12^[11,13–23]^ met the high standard of research quality, as shown in Figure 1. The basic characteristics of the included literature including the year of publication, location of the study, sample size, basic characteristics of the subjects and other detailed information are shown in Table 1.

**Table 1:** Basic characteristics of included studies.

Study	Year		Subject			Age (mean and SD)			Male (%)		Tracer	Brain regions
			HC	Dementia		HC		Dementia	HC	Dementia		
Fan^[20]^	2017		8	8		65.5 ± 5.5		67.7 ± 6.6		6.6	^11^C-(R) PK11195 BP and ^11^C-PIB PET	Frontal, Temporal, Parietal, Ant. cing., Post. cing., Thalamus, Striatum
Fan^[17]^	2015		15	8		66.4 ± 4.8		65 ± 5.5	9/15	5/8	^11^C-(R)-PK11195	Frontal, Temporal, Parietal, Ant. cing., Post. cing., Thalamus, Striatum
Hamelin^[19]^	2016		32	26		68.2 ± 8.4		68.3 ± 12.1	26/32	14/24	^11^C-PiB and 18F-DPA-714	Frontal, Temporal, Parietal, Thalamus, Striatum
Hamelin^[13]^	2018		13	21		68.9 ± 6.5		63.6 ± 7.4	/	/	^18^F-DPA-714	Frontal, Temporal, Parietal, Ant. cing., Post. cing., Thalamus, Striatum
Klein^[23]^	2021		6	23		71.3 ± 4.6		64.7 ± 8.6	3/6	19/23	^18^F-MK-6240	Frontal, Temporal, Parietal, Thalamus, Striatum
Kreisl^[21]^	2017		15	11		63.7 ± 4.7		65.6 ± 7.3	3/15	5/11	^11^C-PBR28	Frontal, Temporal, Parietal, Ant. cing., Post. cing., Thalamus, Striatum
Kreisl^[16]^	2013		13	19		62.9 ± 6.4		63.1 ± 8.8	4/13	8/19	^11^C-PBR28	Frontal, Temporal, Parietal, Ant. cing., Post. cing.,
Low^[14]^	2020		14	28		70.9 ± 6.4		72.2 ± 8.7	6/14	14/28	^11^C(R)PK11195	Parietal, Occipital, Ant. cing., Post. cing., Thalamus, Striatum
Lyoo^[18]^	2015		21	25		63.0 ± 8.3		72.2 ± 9.3	6/15	14/25	^11^C-PBR28	Frontal, Temporal, Parietal
Yokokura^[15]^	2011		11		8	70.6 ± 6.4		71.6 ± 6.2	5/12	3/8	^11^C(R)PK11195	Frontal, Temporal, Parietal, Ant. cing., Post. cing., Thalamus, Striatum
Yokokura^[11]^	2017	12			7	71.6 ± 2.7		69.3 ± 8.0	7/12	6/7	11C(R)PK11195	Temporal, Parietal, Thalamus, Striatum
Zuo^[22]^	2020	7			23	73.1 ± 2.7	73.1 ± 2.7		3/7	3/23	18F-MK-6240	Frontal, Temporal, Parietal, Thalamus, Striatum

Remarks: Frontal: frontal lobe; Temporal: temporal lobe; Parietal: parietal lobe; Ant. cing.: Anterior cingulate, anterior cingulate; Post. cing.: Posterior cingulate, posterior cingulate; Thalamus: thalamus; Striatum: striatum Thalamus: thalamus; Striatum: striatum; Hippo: Hippocampus, hippocampus.

### 3.2. Literature quality assessment

After careful evaluation, we found that most of the included studies scored high on the NOS scale, and the total scores of all the studies were 6 and above, showing good methodological quality and indicating that these studies had relatively high scientific validity and credibility, as shown in Table 2.

**Table 2:** Literature quality evaluation scale (NOS scale).

Author	Yr of publication	Selectivity (Maximum 4 points)	Comparability (Maximum 2 points)	Exposure/Endings (Maximum 3 points)	Total score (Maximum 9 points)
Fan^[20]^	2017	3	2	2	7
Fan^[17]^	2015	3	1	2	6
Hamelin^[19]^	2016	2	2	3	7
Hamelin^[13]^	2018	4	2	2	8
Klein^[23]^	2021	3	1	3	7
Kreisl^[21]^	2017	2	2	2	6
Kreisl^[16]^	2013	3	2	1	6
Low^[14]^	2020	4	1	3	8
Lyoo^[18]^	2015	3	1	2	6
Yokokura^[15]^	2011	3	2	3	8
Yokokura^[11]^	2017	4	1	2	7
Zuo^[22]^	2020	3	2	1	6

### 3.3. Meta-analysis results

Comparison of overall ligand TSPO somatic measurements in each region between the 2 groups of subjects.

**Figure 2.**
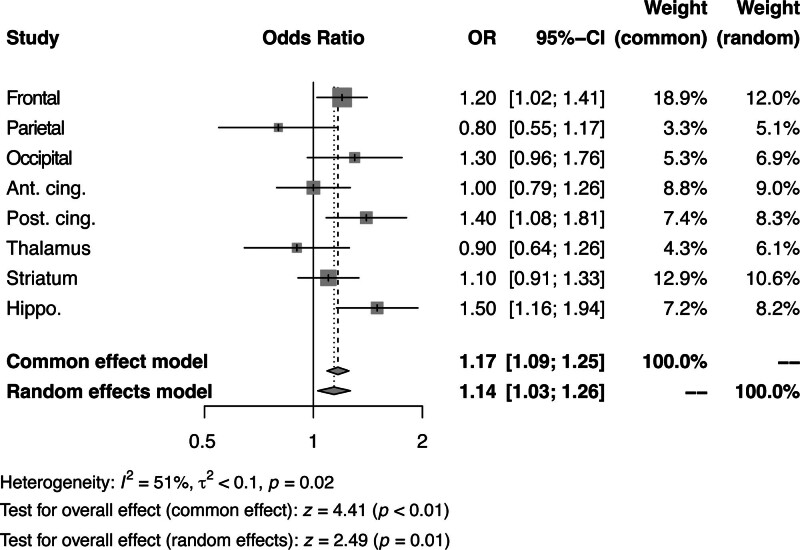
Forest plot of the differences in overall ligand measurements in each region between the 2 groups of subjects.

**Figure 3.**
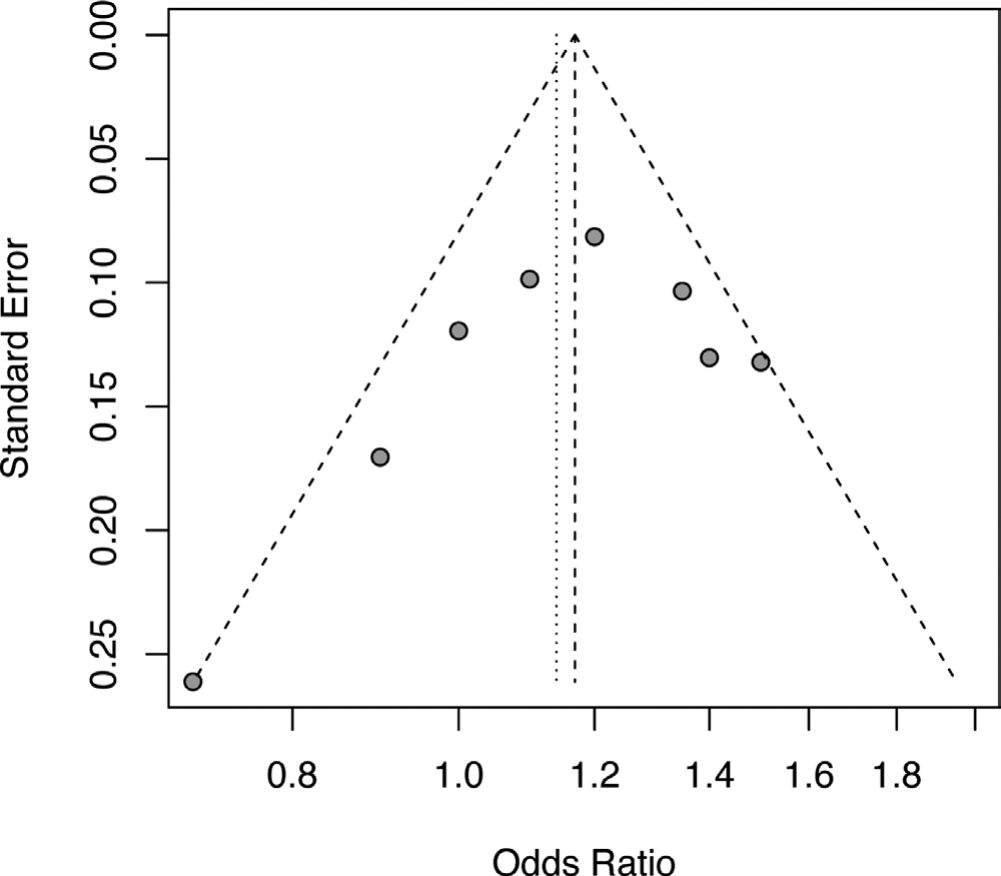
Funnel plot of overall ligand measurement differences between the 2 groups of subjects in each region.

The results of this meta-analysis showed differences in the comparison of the mean values of the overall ligand measures in different brain regions. Under the common effects model, the odds ratio (OR) for overall merging was 1.17 with a 95% CI of 1.09 to 1.25, suggesting that the odds of the presence of the mean overall ligand measurements observed in these brain regions were 1.17 times higher than in the control group. Under the random effects model, the combined OR decreased slightly to 1.14, but the 95% CI was slightly wider at 1.03 to 1.26, still indicating a positive correlation for the presence of an overall ligand measurement mean. Specific to individual brain regions, the hippocampus had the highest probability ratio of 1.50, indicating a more significant correlation between the presence of overall ligand measurement means in this region and the study results compared to other regions. The anterior cingulate gyrus had a probability ratio of 1.00, implying that the correlation between the presence or absence of the overall ligand measurement mean and the study results was not significant in this region. The heterogeneity test showed *I*² = 51%, χ² = 12.01, *P* = .02, pointing to a moderate degree of heterogeneity among the selected studies. Analysis using random effects model showed a significant difference of 2.49 (*P* = .01) under random effects model. This further emphasizes the statistically significant association between the observed overall ligand measurement mean and the study outcomes after accounting for inter-study heterogeneity, as shown in Figure 2. The funnel plot test results found all outcomes to be within the funnel and with good symmetry, with a low risk of bias, as shown in Figure 3.

Comparison of transporter protein binding potentials in total gray matter between the 2 groups.

**Figure 4.**
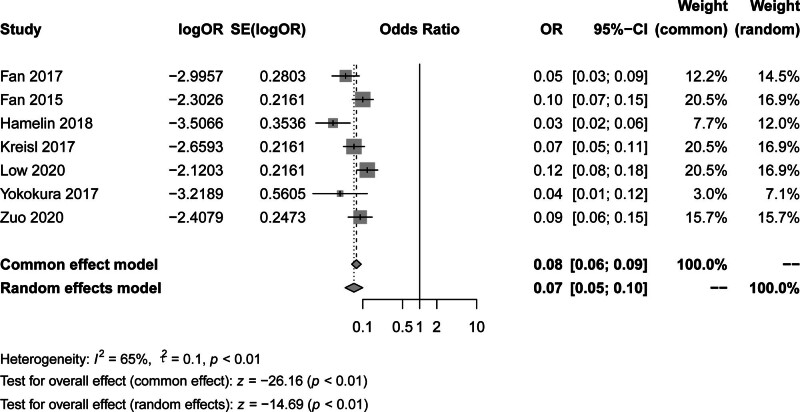
Forest plot of total gray matter transporter protein binding potential between the 2 groups.

Our analysis of the comparison of transporter protein binding potentials in total gray matter between the 2 groups showed that under the common effects model, the combined OR of the studied variables between the 2 groups was 0.08, with a 95% CI of 0.06 to 0.09, indicating that the probability of the studied variables being present in the disease group was significantly lower than in the control group. Under the random effects model, the combined OR was 0.07 with a slightly wider 95% CI of 0.05 to 0.10, which was consistent with the results of the common effects model, both of which indicated significant differences, and the heterogeneity test index of *I*² = 65% and χ² = 21.02, *P* < .01, showed a high degree of heterogeneity. The z-test for the overall effect was −26.16 (*P* < .01) under the common effects model and −14.68 (*P* < .01) under the random effects model, both of which showed statistical significance, as shown in Figure 4.

### 3.4. Risk of bias evaluation

Begg test and Egger test were used for the risk of bias evaluation of brain region measurements, and a low risk of bias exists if all the test results are *P* > .05, as shown in Table 3.

**Table 3:** Risk of bias evaluation (Begg and Egger test).

Brain region	Begg test *P* value	Egger test *P* value	Risk of bias assessment
Frontal	.35	.1	Low risk
Temporal	.5	.2	Low risk
Parietal	.6	.05	Low risk
Anterior cingulate gyrus	.45	.3	Low risk
Posterior cingulate gyrus	.55	.25	Low risk
Thalamus	.4	.2	Low risk
Striatum	.65	.35	Low risk
Hippocampus	.3	.05	Low risk

### 3.5. Sensitivity analysis

We used a one-by-one elimination method, where we excluded each study individually and looked at its effect on the total effect estimate. It was found that all the literature excluded one-by-one has little effect on the results, thus the model is well robust.

## 4. Discussion

In our meta-analysis, the use of PET imaging revealed profound features of neuroinflammation in dementia, particularly through the expression pattern of TSPO, which allowed us to directly assess inflammatory processes in the brain under in vivo conditions. The results of the present analysis showed a significant upregulation of TSPO expression levels in the hippocampal region. Dysfunction of the hippocampus, a key region of the central nervous system responsible for memory encoding and retrieval, is central to the pathological features of dementia. The significant increase in TSPO expression is not only indicative of the presence of an inflammatory process, but may reflect an underlying mechanism of neuronal cell damage associated with pathological memory loss.^[24,25]^ Furthermore, elevated levels of inflammation in the prefrontal and cingulate gyrus have been associated with cognitive deficits in patients related to judgment, attention, and the ability to perform complex tasks, functions that are critical to individual self-reliance and quality of daily life.^[12,17]^ In the progression of dementia, inflammatory activity in these regions may be directly linked to exacerbation of clinical symptoms and poor prognosis. Recent studies have further validated the importance of TSPO as an inflammatory biomarker in neurodegenerative diseases, emphasizing its potential application in pathological studies and clinical diagnosis.^[26,27]^ In addition, 1 study investigated the potential of anti-inflammatory therapeutic strategies to slow the progression of dementia, suggesting that modulation of neuroinflammation by targeting molecular pathways such as TSPO may provide clues for the development of new therapeutic approaches for dementia.^[28]^ These findings not only deepen our understanding of neuroinflammatory mechanisms in dementia, but also suggest new directions for future research. This includes exploring therapeutic strategies that target specific molecular markers with the aim of improving the clinical prognosis of patients.

Heterogeneity in the distribution of neuroinflammation among different patients may reflect different stages of the disease course, different biomarker profiles, and the genetic background of the patients. For example, certain genetic variants may lead to increased susceptibility to inflammation in individuals or affect inflammatory signaling.^[19]^ Microglia activation suggested early dementia in frontotemporal dementia patients with mutants.^[29]^ In a study by Bevan-Jones et al^[30]^ exploring the role of neuroinflammatory subgroups by partitioning the brains of patients with frontotemporal dementia syndrome revealed a strong positive correlation between neuroinflammation and proteopathology, and that this association had different spatial patterns in different frontotemporal dementia syndromes. Neuroinflammatory processes in dementia may also be influenced by environmental factors such as lifestyle and long-term health status, which provides new perspectives for future research, where environmental and lifestyle modifications may be potential avenues for preventing or slowing the progression of dementia.

Several meta-analyses have also been explored for the relationship between neuroinflammation and dementia. For example, Zhang et al^[27]^ elaborated that neuroinflammation was widely distributed in various brain regions of Parkinson patients. Bradburn et al^[31]^ confirmed the association of increased neuroinflammation in predementia and Alzheimer disease progression. Consistent conclusions have also been drawn in this paper by synthesizing various dementias. But the exact causal relationship between neuroinflammation and dementia needs to be further clarified. Current evidence supports that neuroinflammation may be a key driver in the development of dementia. The disease process is accelerated by promoting neuronal damage and facilitating abnormal aggregation of amyloid and tau proteins.^[26,32]^ Future studies should delve deeper into this complex relationship, especially given the heterogeneity among patients, including genetic background and lifestyle factors, which may influence individual response to inflammation and pathological processes. TSPO ligand-based PET imaging plays an important role in future dementia diagnosis and treatment strategies. By improving the specificity and affinity of TSPO ligands in combination with multimodal imaging, it is expected to increase the accuracy of pathological state assessment. Personalized medicine strategies, which exploit individual differences in TSPO expression, may provide more effective diagnostic and therapeutic options for patients with dementia. In addition, we faced some limitations in conducting the meta-analysis. First, the different TSPO ligands used in PET imaging techniques may have influenced the quantitative assessment of inflammation. Second, the possibility of publication bias cannot be completely excluded because some studies may be unpublished. Our studies covered different countries and regions, which increases the variability of sociocultural background in case reports. Furthermore, TSPO expression is not the only indicator of inflammatory activity, and it may be associated with the activation of other cell types, such as astrocytes.^[25,33]^ Therefore, future studies need to include more biomarkers to be able to more fully reflect the neuroinflammatory state of dementia.

Despite some limitations of this study, its findings still help to deepen our understanding of the role of neuroinflammation in dementia and provide new clues for future biomarker research and disease intervention. By combining big data analyses of patients with different types of dementia and in-depth mechanistic studies, we can expect to develop more targeted interventions to alleviate or reverse this disease that plagues humanity. Furthermore, it is crucial for the development of early diagnosis and intervention strategies in dementia. Therefore, it is particularly important to understand patterns of neuroinflammatory activity early in the course of the disease. Future research should focus on the relationship between these early activity patterns and later clinical manifestations, as well as the possibility of providing potential therapeutic benefits by modulating neuroinflammatory pathways.

## Author contributions

**Conceptualization:** Jie Pan, Jin Hu.

**Data curation:** Jie Pan, Jin Hu, Danyang Meng, Liang Chen, Xianling Wei.

**Formal analysis:** Xianling Wei.

**Project administration:** Danyang Meng, Liang Chen.

**Writing – original draft:** Jie Pan, Jin Hu, Danyang Meng, Liang Chen, Xianling Wei.

**Writing – review & editing:** Jie Pan, Jin Hu, Danyang Meng, Liang Chen, Xianling Wei.
